# Safety and efficacy of tocilizumab in children with systemic juvenile idiopathic arthritis

**DOI:** 10.1186/1546-0096-13-S1-P165

**Published:** 2015-09-28

**Authors:** G Horneff, I Huppertz, P Haas, K Minden, G Ganser, A Hospach, R Trauzeddel

**Affiliations:** 1 Asklepios Clinic, Sankt Augustin, Germany; 2 Prof.-Hess-Kinderklinik, Bremen, Germany; 3 Deutsches Zentrum für Kinder- und Jugendrheumatologie, Garmisch-Partenkirchen, Germany; 4 Deutsches Rheuma-Forschungszentrum (DRFZ), Berlin, Germany; 5 Sankt Josef Stift, Sendenhorst, Germany; 6 Olga Hospital, Stuttgart, Germany; 7 Klinik für Kinderheilkunde u. Jugendmedizin, Helios Klinikum, Berlin, Germany

## Background

Since its approval in 2011 for treatment of systemic juvenile idiopathic arthritis (JIA), tocilizumab treatments are followed by the German biologics register (BiKeR). The aim of this interim analysis is to evaluate the efficacy and safety of tocilizumab under practical conditions in childhood.

## Methods

Demographics, clinical characteristics, previous and concomitant therapy, parameters of disease activity and adverse events were documented prospectively. The efficacy was based on the PedACR 30/50/70/90 criteria and the JADAS10. Tolerability was captured by the primary treating physician to the adverse event reports.

## Results

Until 31.12.2014 60 sJIA patients were included in BiKeR in which treatment with tocilizumab was started. The mean age at onset was 4.6 (median 3.3) years and the mean age at baseline was 9.4 (median 9.8) years and mean disease duration was 4.7 (median 3.6) years. Only 20.3% of patients were treated with tocilizumab in the first two years of their disease. Pre-treatment with NSAIDs was carried out at 52 (86.7%), with steroids in 58 (96.7%), with methotrexate in 53 (88.3%). 26 treatment attempts with other DMARDs, most commonly with azathioprine (9; 15%) or CSA (9; 15%) and 44 therapeutic trials with other biologics were made. Etanercept was used most frequently (25; 41.7%) followed by anakinra (23.3%; 14). Concomitant therapy consisted of NSAIDs (38; 63%), steroids (42; 70%) and methotrexate (38; 63.3%) with other DMARDs only in individual cases.

Most patients showed a significant response to treatment. At last documentation 62% / 58% / 50% reached a JIA ACR30/50/70 response. The mean JADAS10 showed a decrease from 17.5 to 6.0 / 3.0 / 4.0 / 3.0 / 4.0 after 3/6/12/18/24 months. The proportion of patients in remission (JADAS10≤1) at month 6, 12, 24 was 42% / 25% / 27%, and the proportion in JADAS minimal disease actvity, MDA (JADAS10≤3,8) 55%, 35% and 55%.

Until 31.12.2014 a total of 74 adverse events (AE) were reported (101.7 / 100 patient-years (CI 81.0 to 127.8)), of which 10 (13.7/100 patient-years (CI 7.4 to 25, 5)) were serious (SAE). Infections were reported most frequently with 33 events. 5 AEs related cytopenias (without MAS), 4 intolerance reactions. 3 infections were SAE (appendicitis, pneumonia, herpes zoster). 4 patients developed a macrophage activation syndrome. Further SAE occurred once, anaphylaxis, seizure, fracture. Opportunistic infections, including tuberculosis, malignancies or deaths were not reported. In 32 patients (53.3%) the treatment was stopped. Reasons were (several simultaneously possible) remission in 15 (25%), ineffectiveness 7 (11.7%), patient request 5 (8.3%), intolerance 4 (6.7%) other 1 (1.7%).

## Summary

Upon therapy with tocilizumab a high ACR response was achieved by many sJIA patients, as well as a JADAS MDA. A JADAS remission was documented in up to 40% of patients. In many patients, tocilizumab was used late and as second biologic. The tolerability was good overall and comparable to those of other biologics in JIA. Only a few patients discontinued therapy because of intolerance or side effects.

